# Pathways to prevention: the Accelerating Medicines Partnership® Schizophrenia (AMP® SCZ) Program

**DOI:** 10.1038/s41537-025-00605-1

**Published:** 2025-04-15

**Authors:** Barnaby Nelson, Martha E. Shenton, Scott W. Woods, the Accelerating Medicines Partnership® Schizophrenia (AMP® SCZ), Barnaby Nelson, Martha E. Shenton, Scott W. Woods

**Affiliations:** 1 Centre for Youth Mental Health, The University of Melbourne, Parkville, Victoria, Australia; 2 Orygen, Parkville, Victoria, Australia; 3 Harvard Medical School, Boston, MA USA; 4 Mass General Brigham, Founding Member, Brigham and Women’s Hospital, Boston, MA USA; 5 Yale University, New Haven, CT USA

**Keywords:** Psychosis, Biomarkers

Watch Drs. Barnaby Nelson and Scott W. Woods discuss their work https://vimeo.com/1065077703?share=copy#t=0 and Dr. Martha E. Shenton discuss her work https://vimeo.com/1065077556?share=copy#t=0.

Signs and symptoms of risk for developing schizophrenia and other psychotic disorders are generally present months to years before illness onset. They include attenuated psychotic symptoms, decline in social and role functioning, and a range of non-specific symptoms that overlap with other psychiatric illnesses. This stage of potential illness has been operationalized as the clinical high risk (CHR) syndrome for psychosis.

CHR is a common condition, with a prevalence estimated in the general youth population of 1.7% and 19.2% in youth at their first presentation for psychiatric treatment^1^. Moreover, at least 78% of individuals who develop schizophrenia or other psychotic disorders experience a prodromal period similar to CHR^2^. Longitudinal studies of CHR individuals suggest an estimated 20% conversion to psychosis at two-year follow-up^3^ and an estimated 41% remission from the CHR syndrome^4^. However, this latter sub-group may have persistent or new onset non-psychotic symptoms and disorders^5^. The remainder shows progressing or persisting attenuated psychotic symptoms^6^, impaired functioning^4^, cognition^7^ and other symptoms^4^.

While there is evidence that treatment during this stage is beneficial^8,9^, more effective and personalized interventions are needed to reduce or halt psychosis risk and other adverse clinical outcomes, as noted above, such as persistent attenuated psychotic symptoms, non-psychotic symptomatology, and poor functioning. There are also currently no medications approved by regulatory agencies for this clinical group, despite the clear clinical need and the opportunity for preventative treatment. Clinicians need further guidance on what treatments to use, in which sequence, and how to tailor the treatment to the individual CHR patient. Treatment development can be facilitated by generating tools informed by mechanistic processes that identify the level of risk for psychosis conversion and other potential treatment targets. These tools can be used to stratify CHR individuals for future clinical trials with novel therapeutics and to develop personalized treatments for each individual.

The Accelerating Medicines Partnership^®^ Schizophrenia (AMP^®^ SCZ) was established in 2020 to achieve these aims^10,11^. The AMP program was initiated by Francis Collins in 2014, then director of the National Institutes of Health (NIH). He went before Congress to create an unprecedented public-private effort to identify, develop, and fast-track treatment targets in areas of high unmet need.

The list of AMP projects is wide-ranging. There are now nine such projects currently underway (Alzheimer’s Disease 2.0, Amyotrophic Lateral Sclerosis, Autoimmune and Immune-Mediated Disease, Bespoke Gene Therapy Consortium, Common Metabolic Disorders, Heart Failure, Parkinson’s Disease, Parkinson’s Disease and Related Disorders, and Schizophrenia) and three completed projects (Alzheimer’s Disease 1.0, Type 2 Diabetes, and Rheumatoid Arthritis/Systemic Lupus Erythematosus)^12^.

The AMP SCZ project is an important large international collaboration to develop algorithms using a set of clinical and cognitive assessments, multimodal biomarkers (EEG, MRI, fluid biospecimens, speech, and digital), and clinical endpoints. The latter, clinical endpoints, can then be used to predict the trajectories and outcomes of CHR individuals, with the ultimate goal of advancing treatment development for this clinical population. An underlying premise is that early intervention for schizophrenia and other psychoses is a critical component for achieving meaningful improvement in the treatment of these conditions. This focus on CHR will lead to drug development tools that will be used to stratify individuals for clinical trials and will serve as early indicators of treatment efficacy. These tools will bring us closer to other areas of medicine that use medical devices and other tools to facilitate treatment efficacy and to assess outcomes. The size, scope, and design of the consortium address the limitations of previous CHR observational studies, such as modest statistical power related to small sample sizes, lack of a validation sample, a narrow set of measures (which limits inference regarding the relationship between data modalities and the development of multimodal prediction models), and lack of harmonized assessment tools.

The consortium, managed by the Foundation for the National Institutes of Health (FNIH), unites diverse expertise, including scientific, regulatory, and lived experience. Participants include the US National Institute of Mental Health (NIMH), US Food and Drug Administration (FDA), European Medicines Agency (EMA), private industry (Boehringer Ingelheim, Janssen Research & Development, Otsuka Pharmaceutical Development & Commercialization), non-profit and patient advocacy organizations (American Psychiatric Association Foundation, National Alliance on Mental Illness, One Mind, Schizophrenia & Psychosis Action Alliance), and the Wellcome Trust charitable foundation. The AMP SCZ project has been introduced to the field in two previous publications^10,11^. The current collection of papers aims to explore each domain in detail by highlighting its importance for achieving AMP SCZ’s goals, its potential contribution to the broader field, the rationale and details for measurement selection, assessment procedures, and some preliminary data from baseline measures.

The AMP SCZ program is composed of two Research Networks – the Psychosis Risk Outcomes Network (ProNET), led by Yale University, and the Trajectories and Predictors in the CHR for Psychosis Population: PREdiction SCIENTific Global Consortium (PRESCIENT), led by Orygen/The University of Melbourne – and the Psychosis Risk Evaluation, Data Integration and Computational Technologies: Data Processing, Analysis, and Coordination Center (PREDICT-DPACC), led by Brigham and Women’s Hospital, Harvard Medical School.

The data collected by the two Research Networks is fully harmonized for the assessment batteries and study protocols, and extensive multimodal data are being collected from CHR individuals (*n* = 1977) and healthy controls (*n* = 640), with a follow-up period of two years. Recruitment reached 100% of target enrolment in January 2025. Together, the research networks consist of 43 sites across 14 countries and 5 continents. As detailed in the clinical domain paper in this collection^13^, CHR ascertainment criteria are harmonized for use in the consortium using the Positive SYmptoms for CAARMS Harmonized with SIPS (PSYCHS) interview, a new psychometric instrument for defining CHR, the severity of attenuated psychotic symptoms, and conversion to psychosis^14^.

The DPACC is responsible for managing, processing, disseminating, archiving, and analyzing the AMP SCZ data. As detailed in the Data Flow^15^ and Dissemination^16^ papers in this collection, the project is guided by open science principles, with data quality assessment/control and rapid dissemination, making the data accessible to all qualified researchers in the general research community through the NIMH Data Archive (NDA). The first data release in November of 2023 consisted of 430 participants, the second data release in June of 2024 consisted of 1048 participants, and the third data release in January of 2025 consisted of 2193 participants.

CHR and HC participants are assessed with a core set of measures at baseline and month 2 follow up, with follow up assessments occurring at regular intervals over the follow up period, including monthly symptom ratings for the first year. The assessment battery was carefully developed through an extensive process of team consultation, with expert representatives across the research networks, PREDICT-DPACC, AMP SCZ partners, and NIMH. The evidence base, innovation, feasibility, and participant/rater burden were key considerations in the development of the battery, as detailed in the accompanying papers. Measures include clinical^13^ and cognitive^17^ assessments; neurophysiology^18^, neuroimaging^19^, genetics and fluid biomarkers^20^; speech and facial expression (audio/video recording)^21^; and digital assessments^22^. With regard to the latter, the digital assessments collect active (e.g., daily survey on social interactions and feelings of connectedness) and passive data (e.g., sleep time, number of texts and phone calls; time spent in green space, home, school, exercising, therapy visits, and social relationships), along with an automated assessment of social and community functioning from the global positioning system (GPS) data. The digital and clinical measures will be important for capturing extensive biopsychosocial data relevant to clinical trajectories, which could then be targeted in psychosocial interventions. The papers also present some preliminary analysis of the data collected to date highlighting the potential of this data set^13,17–19,21^. As detailed by Asgari-Targhi et al.^16^ lived experience of psychosis is embedded in every facet of the program.

The primary endpoint of interest is conversion to psychosis by 12 and 24 month follow-up. Secondary clinical endpoints of interest include remission from CHR syndrome, and nonconversion/nonremission. Clinical outcomes of interest span multiple domains, including attenuated positive symptoms, mood and anxiety, psychosocial functioning, and persistent negative symptoms.

The biomarker data collected across the research networks will be analyzed by the DPACC to create drug development tools from multimodal prediction models and risk calculators by drawing on recent theoretical and methodological advances (e.g., dynamic prediction, probabilistic multimodal modeling) and informed by the analysis of legacy datasets^23^. These tools will guide the selection and stratification of CHR individuals for future clinical trials based on the trials’ primary endpoints of interest. For example, stratification can identify a subset of CHR individuals who are at higher risk for developing psychosis relative to the rest, providing an enriched sample for testing new pharmacological treatments. Similarly, trials could be enriched for attenuated positive symptom severity or a biomarker that predicted absence of decline in attenuated positive symptom severity in the observational study. These models will also facilitate the testing of more precise and mechanism-guided treatments. The first of the trials under the AMP SCZ umbrella was funded in 2024.

The tools may also have immediate clinical utility in decision-making about stepping interventions up or down as risk is assessed over time (capturing clinical trajectory and treatment response) and in response to incoming biomarker information. Some tools, such as the risk calculators, will prioritize the more readily acquired clinical data and biomarkers for prediction to enable clinical tools (proxies for the biomarkers) that may be more feasible to implement in community-based and non-specialized settings.

By integrating the strengths of multiple international stakeholders, bringing together experienced researchers in this field, sharing discoveries openly, and priming future research, the AMP SCZ program will catalyze discovery about psychosis risk and advance preventive intervention at the earliest stages of schizophrenia. The papers in this collection provide the reader with a roadmap to the breadth and depth of what promises to be a landmark dataset for use by all qualified researchers in the general research community via the NIMH Data Archive.

## Supplementary information

AMP SCZ Member List & Affiliations
